# Grain Boundaries Contribute to the Performance of Perovskite Solar Cells by Promoting Charge Separations

**DOI:** 10.1007/s40820-025-01795-0

**Published:** 2025-06-04

**Authors:** Peng Xu, Pengfei Wang, Minhuan Wang, Fengke Sun, Jing Leng, Yantao Shi, Shengye Jin, Wenming Tian

**Affiliations:** 1 Department of Chemical Physics, University of Science and Technology of China, Hefei, 230026 People’s Republic of China; 2 State Key Laboratory of Chemical Reaction Dynamics, Dalian Institute of Chemical Physics, Chinese Academy of Sciences, Dalian, 116023 People’s Republic of China; 3 State Key Laboratory of Fine Chemicals, School of Chemistry, Frontier Science Center for Smart Materials, Dalian University of Technology, Dalian, 116024 People’s Republic of China; 4 Key Laboratory of Materials Modification By Laser, Ion and Electron Beams (Ministry of Education), School of Physics, Dalian University of Technology, Dalian, 116024 People’s Republic of China; 5 University of Chinese Academy of Sciences, Beijing, 100049 People’s Republic of China

**Keywords:** Perovskite solar cells, Grain boundary, Photocurrent mapping, Stark effect, Carrier dynamics

## Abstract

Historically seen as a limitation, grain boundaries (GBs) within polycrystalline metal halide perovskite (MHP) films are thought to impede charge transport, adversely impacting the efficiency of perovskite solar cells (PSCs). In this study, we employ home-built confocal photoluminescence microscopy, combined with photocurrent detection modules, to directly visualize the carrier dynamics in the MHP film of PSCs under real operating conditions. Our findings suggest that GBs in high-efficiency PSCs function as carrier transport channels, where a notable enhancement in photocurrent is observed. Femtosecond transient absorption and Kelvin probe force microscopy measurements further validate the existence of a built-in electric field in the vicinity of GBs, offering additional driving force for charge separation and establishing channels for swift carrier transport along the GBs, thereby expediting subsequent charge collection processes. This study elucidates the pivotal role of GBs in operational PSCs and provides valuable insights for the fabrication of high-efficiency PSCs.

**Supplementary Information:**

The online version contains supplementary material available at 10.1007/s40820-025-01795-0.

## Highlights

- Sub-micrometer-resolved photocurrent mapping in operational perovskite solar cells, achieved through our home-built photoluminescence and photocurrent imaging microscopy, reveals enhanced photocurrent at grain boundaries compared to grain interiors.
- Local pump-probe femtosecond transient absorption and Kelvin probe force microscopy measurements corroborate the presence of a built-in electric field in the vicinity of grain boundaries that promotes electron–hole separation and the subsequent charge collection, thereby contributing to the performance of perovskite solar cells.

**Supplementary Information:**

The online version contains supplementary material available at 10.1007/s40820-025-01795-0.

## Graphical abstract

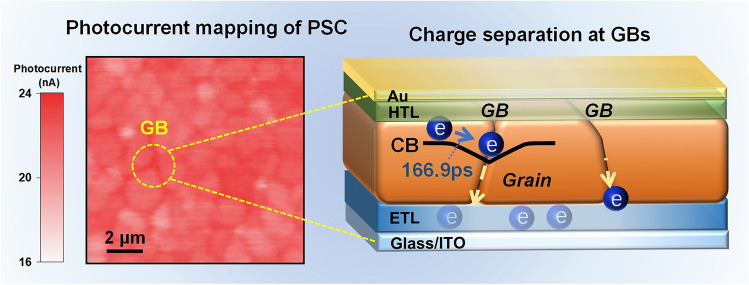

## Introduction

Polycrystalline semiconductor films are widely utilized in a variety of devices, including solar cells [1–6], thin-film transistors (TFT) [7–10], thin-film thermoelectric generators [11, 12], and microelectromechanical systems (MEMS) [13]. The performance of these devices hinges on the polycrystalline film's microstructures, particularly grain boundaries (GBs) [14]—the interfaces that separate individual grains oriented in distinct crystallographic directions [15]. Metal halide perovskite solar cells (PSCs) have achieved fast progress in power conversion efficiency (PCE), which is largely determined by the quality of the metal halide perovskite (MHP) polycrystalline film that contains a large quantity of GBs [16, 17]. Comprehending the role of GBs in polycrystalline MHP film is imperative for the rational design of the active layer and, ultimately, for enhancing device performance [18–20].

In the realm of PSCs, the GBs in MHP films have predominantly been labeled as detrimental factors that influence device stability [21, 22] and efficiency [17, 23–25]. This perspective is primarily attributed to the markedly greater defect density at GBs in comparison with the bulk phase [23]. It is widely accepted that GBs inherently hinder charge transport through their scattering effects or trapping mechanisms, consequently exacerbating nonradiative recombination and leading to energy loss [25, 26]. However, PSCs using single-crystal MHP films or those with ultra-large grains did not achieve higher PCE as anticipated, suggesting that the impact of GBs may not be entirely negative [27, 28]. Actually, in other solar cells such as polycrystalline CdTe [6] and copper indium gallium selenide (CIGS) [29], certain specific GBs have been found to paradoxically enhance the efficiency of charge transport. Recent studies have proposed that GBs in MHP films may enhance the efficiency of PSCs by facilitating the separation of electrons and holes [30, 31] However, this conclusion was drawn from the investigation on isolated MHP films, rather than on fully assembled or operational PSCs. Therefore, the role of GBs (such as whether they facilitate carrier transport as in CdTe or CIGS solar cells) is indeed unclear in an operational PSC.

In this work, we employ home-built confocal photoluminescence (PL) microscopy, combined with a photocurrent detection module, to directly map the local photocurrent and PL in operational PSCs with different PCE values with a sub-micrometer spatial resolution. We find that the local photocurrent at the GBs is notably higher than the inside of grains and exhibits an inverse correlation with the local GB PL intensity. Furthermore, the high-efficiency PSCs show a greater amplitude of photocurrent enhancement at GBs compared to the low-efficiency PSCs. Local pump-probe femtosecond transient absorption and Kelvin probe force microscopy (KPFM) measurements corroborate the presence of a built-in electric field in the vicinity of GBs that promotes electron–hole separation and the subsequent charge collection at GBs. Conversely, a high density of defects at GBs can trap carriers, leading to performance degradation, as observed in low-efficiency PSCs. This research clarifies the dual role of GBs in PSCs, emphasizing their beneficial impact on high-efficiency devices, which will significantly contribute to enhancing the performance of PSCs.

## Experimental Section

### Materials

The SnO_2_ colloid (tin (IV) oxide) precursor (15 wt% in H_2_O colloidal dispersion) was obtained from Alfa Aesar. Ndimethylformamide (DMF), dimethyl sulfoxide (DMSO, 99.9%), acetonitrile (ACN, 99.8%), methylbenzene (99.9%), chlorobenzene (CB, 99.8%) and Bis (trifluoromethane) sulfonimide lithium salt (Li-TFSI, 99.95% trace metals basis) were obtained from Sigma-Aldrich. Lead iodide PbI_2_ (> 99.999%) and Formamidinium iodide (FAI) (99.8%) were obtained from Advanced Election Technology Co., Ltd. Methylammonium bromide (MABr) (99.9%) and methylammonium chloride (MACl) (99.9%) were purchased from Xi′an Polymer Light Technology. 2,2,7,7′-tetrakis (N,Ndip-methoxyphenylamine)-9,9′- spirobifluorene (Spiro-OMeTAD, 99.8%) was purchased from Borun New Material Technology. Au and Ag were purchased from ZhongNuo Advanced Material (Beijing) Technology Co., Ltd. All the chemicals were used as received without further treatment.

### Fabrication of Perovskite Solar Cells and Perovskite Thin Films

#### Device Fabrication

The ultra-thin ITO (0.15 mm) substrates were washed with deionized water, ethanol and isopropanol successively and were dried under nitrogen flow. After 15 min of UV-ozone treatment, the SnO_2_ colloidal dispersion was diluted to 2.5 wt% with deionized water. Subsequently, the prepared SnO_2_ solution was spun onto the blow-dried ultra-thin ITO substrate with 3000 r min^−1^ for 30 s and heated at 150 °C for 30 min in ambient air to prepare the electron-transport layer (ETL), then reserved 5 min UV-ozone treatment and transferred to the N_2_ glove box. The (FAPbI_3_)_0.95_(MAPbBr_3_)_0.05_ precursor solution was prepared by adding FAI (274.46 mg), PbI_2_ (735.77 mg), MABr (9.41 mg), PbBr_2_ (30.83 mg) and MACl (30.83 mg) into 1.2 mL of a mixed solvent of DMF and DMSO (7:1 by volume) stirred at room temperature for 6 h. To prepare the metal halide perovskite (MHP) film, 50 μL of above perovskite precursor was spread on the ITO/SnO_2_ substrates and spun by a two-stage spin-coating process (1000 r min^−1^ for 10 s and 5000 r min^−1^ for 30 s). During the second spin coating stage, 150 μL of methylbenzene was continuously dripped on the spinning substrate 15 s prior the end of the program. The films were then transferred to a hot plate and annealed at 120 °C for 5and 20 min. Precursor solution of HTL was prepared by dissolving 72.3 mg spiro-OMeTAD, 28.8 μL 4-tert-butylpyridine, 17.5 μL lithium bis (trifluoromethylsulphonyl) imide acetonitrile solution (520 mg mL^−1^), and 20 μL FK209 acetonitrile solution (300 mg mL^−1^) into 1 mL CB. The HTL solution was then deposited on top of the perovskite layer by spin coating at 3,000 r min^−1^ for 30 s. Finally, an 80 nm Au electrode was deposited by thermal evaporation on top of the HTL.

#### ***Preparation of High-Quality and Low-Quality (FAPbI***_***3***_***)***_***0.95***_***(MAPbBr***_***3***_***)***_***0.05***_*** Perovskites Thin Film***

The distinction between high-quality and low-quality perovskite films in this study was achieved solely by modulating the annealing time, while all other fabrication parameters remained identical. Both films were prepared using the same precursor solution and spin-coating conditions. The (FAPbI_3_)_0.95_(MAPbBr_3_)_0.05_ precursor solution was prepared by adding FAI (274.46 mg), PbI_2_ (735.77 mg), MABr (9.41 mg), PbBr_2_ (30.83 mg) and MACl (30.83 mg) into 1.2 mL of a mixed solvent of DMF and DMSO (7:1 by volume) stirred at room temperature for 6 h. 50 μL of above perovskite precursor was spread on the glass substrates and spun by a two-stage spin-coating process (1000 r min^-1^ for 10 s and 5000 r min^-1^ for 30 s). During the second spin coating stage, 150 μL of methylbenzene was continuously dripped on the spinning substrate 15 s prior the end of the program. High-quality films were synthesized by annealing the sample at 120 °C for 20 min in a nitrogen atmosphere. In contrast, low-quality films resulted from a shorter annealing duration of 5 min.

## Results and Discussion

### PL and Photocurrent Mapping in Operating PSCs

Although the role of GBs in charge transport within MHP films in PSCs is still uncertain, it is expected that their influence may vary in devices exhibiting different PCE levels. The annealing time significantly influences the crystallinity of MHP film, giving rise to grains with different size distributions [32, 33]. This, in turn, results in the formation of GBs with different characteristics (such as the density of defects) that affect the photovoltaic performance of PSCs. We fabricated PSCs with different PCEs by manipulating the annealing time of MHP film. In comparison with the MHP film annealed for 20 min under optimized conditions, the film annealed for 5 min shows lower PL intensity and a shorter PL lifetime (Fig. S1), suggesting inferior crystallinity and a higher density of defects. This is further supported by the XRD (Fig. S2) and SEM characterizations (Fig. S3). Cross-sectional SEM images confirm that the GBs are predominantly vertically aligned from the substrate to the surface (Fig. S4). XRD patterns display incomplete transformation of PbI_2_ and insufficient growth of MHP annealed for 5 min, while SEM images reveal noticeable macroscopic defects, with a few voids visible [34]. The two different MHP films were fabricated into PSCs for further characterization under operational conditions.

Recently, concentrated efforts have been made to measure photoresponse at the micro–nano-scale in perovskite thin films through scanning imaging methods such as conductive-AFM (C-AFM) [30], photoconductive AFM (PC-AFM) [35], and KPFM [6, 36]. However, these techniques depend on a tip-scanning process and require direct contact between the tip and the active layer, posing challenges for the measurement of local photovoltaic parameters in operating PSCs.

**Fig. 1:**
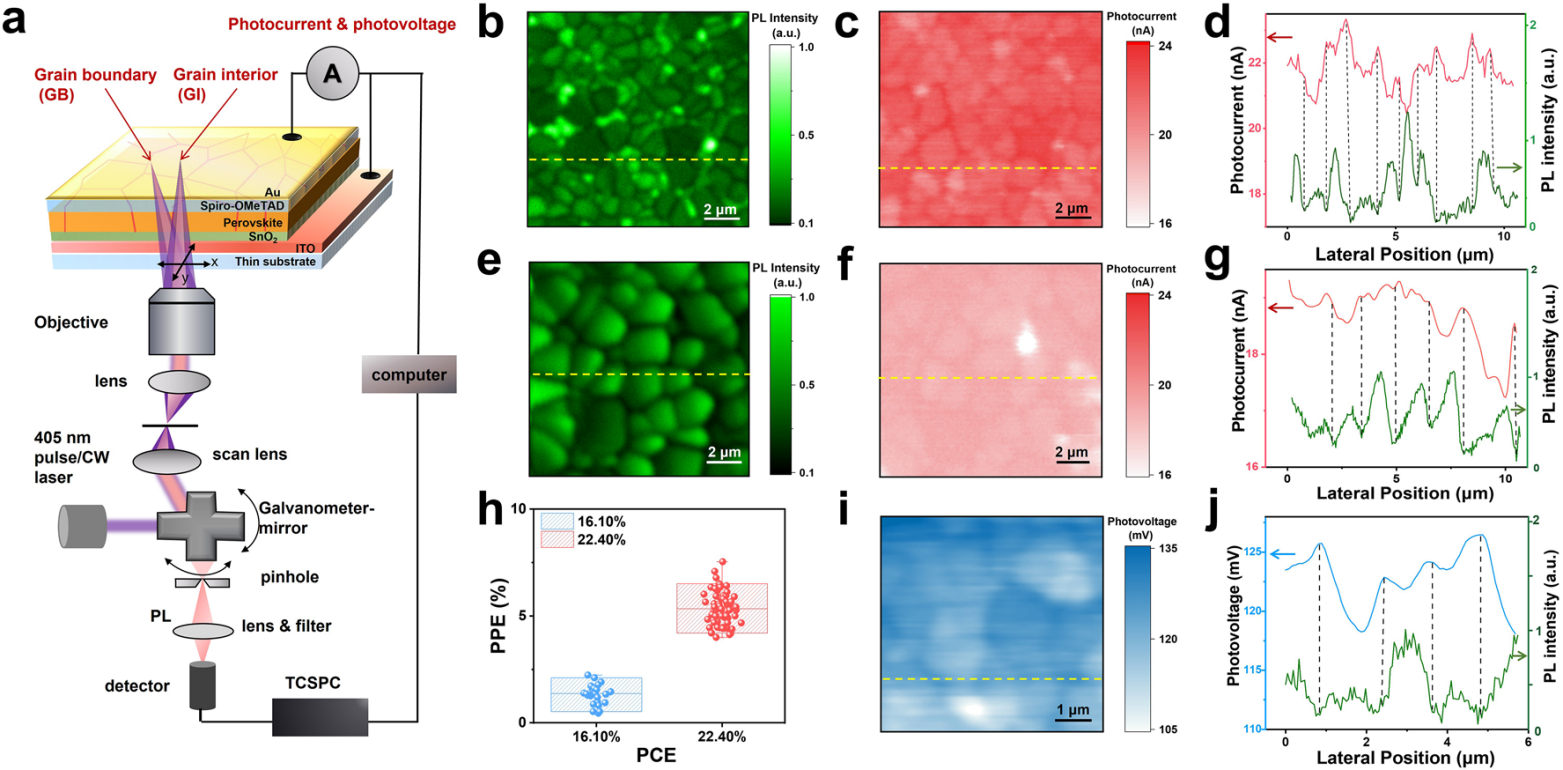
**a** Schematic presentation of the laser-scanned and time-resolved PL microscopy coupled with a photocurrent detection module. This spatial resolution of PL and photocurrent imaging is 260 nm and 500 nm, respectively. **b** Confocal PL intensity image of a PSC with PCE of 22.40%. **c** Photocurrent image on the same area as in **b**. **d** Comparison of the one-dimensional profiles of photocurrent amplitude and PL intensity extracted from the yellow cross lines in **b** and **c**. **e** Confocal PL intensity image of a PSC with PCE of 16.10%. **f** Photocurrent image on the same area as in **e**. **g** Comparison of the one-dimensional profiles of photocurrent amplitude and PL intensity extracted from the yellow cross lines in **e** and **f. h** Statistical diagram of the photocurrent enhancement at GBs for PSCs with PCE of 16.10% and 22.40%, respectively. **i** Photovoltage image of a PSC with PCE of 22.29%. **j** Comparison of the one-dimensional profiles of photovoltage amplitude and PL intensity extracted from the yellow cross lines in **i** and Fig. S11

As an alternative, we employed a home-built setup of laser-scanned PL microscopy coupled with a photocurrent detection module to collect PL and photocurrent on a working PSC, as depicted in Fig. 1a. Briefly, the PSC is excited by a focused laser beam through a 100 × objective. The PL signal is detected by a single-photon detector coupled with a time-correlated single photon counting (TCSPC) module. Simultaneously, the photocurrent and photovoltage signal generated by focused excitation is monitored using a picoammeter. By scanning the laser beam across the PSC via galvanometer mirror rotation, PL, photocurrent and photovoltage images are acquired [37]. We use an autofocus system to dynamically stabilize the focal plane during the measurement. To obtain high-resolution photocurrent and PL mappings, we employed a thin ITO-coated glass (0.15 mm in thickness) for the PSCs fabrication. The spatial resolution of the photocurrent mapping and confocal PL measurement is  ~ 500 and  ~ 300 nm (Fig. S5), respectively. The device architecture and performance of the two PSCs utilizing MHP films with different annealing times (5 and 20 min) are illustrated in Fig. S6, showcasing PCEs of 16.10% and 22.40%. The two devices undergoing subsequent characterizations are designated as the low-efficiency and high-efficiency PSCs, respectively.

PL mapping is an effective method to resolve the microstructures of the perovskite film (Fig. S7). The PL intensity and photocurrent images were collected over an area of 10.5 μm × 10.5 μm for the MHP films in operational PSCs. In the high-efficiency PSCs, the PL intensity image (Fig. 1b) unveils a heterogeneous grain distribution along with clearly defined grain and GB structures. In contrast to the intensity observed on the grains, a notable reduction in PL intensity is noted at the GBs, possibly stemming from either defect trapping or effective charge extraction mechanisms [31, 38]. Following the PL measurement, a corresponding photocurrent image of the same region was acquired (Fig. 1c), which distinctly showcases the microstructures of the grains and GBs, aligning morphologically with the PL intensity image (Fig. 1b). In contrast to the PL intensity distribution, the photocurrent at GBs displays relatively higher intensity than that observed over the grains. To unveil the correlation between photocurrent and PL intensity distributions, we juxtapose the PL profiles with the photocurrent data acquired from the cross-sectional lines in the images of Fig. 1b, c. Figure 1d displays a pronounced anticorrelation between PL and photocurrent intensity, particularly at GBs. The elevated photocurrent and diminished PL intensity at GBs validate their beneficial role in charge separation and carrier transport. To determine the existence of photocurrent enhancement at the grain boundaries under the device's actual operating conditions, we utilized a large-area excitation source of continuous white light, designed to mimic the operational state of the device (Fig. S8). The spatially resolved photocurrent mapping demonstrates consistent spatial characteristics between operational and non-operating states (Fig. S9). Moreover, we investigated the correlation between photocurrent enhancement and power density, with the results presented in Fig. S10. Our results showed that the observed photocurrent enhancement was consistent under different power densities. Detailed analysis and discussion are provided in supporting information.

We also examined whether GBs also exhibit a positive impact on low-efficiency PSCs. Although the grains and GBs are distinctly visible in the PL intensity image (Fig. 1e), distinguishing between the grains and GBs in the photocurrent image poses a challenge (Fig. 1f). Unlike the results in high-efficiency PSCs, the enhancement in photocurrent at GBs in the low-efficiency PSCs is not pronounced and PL intensity and photocurrent are not clearly anti-correlated (Fig. 1g). This phenomenon is ascribed to the presence of a high density of defects that trap carriers in the low-efficiency PSC. We further calculated the percentage of photocurrent enhancement (PPE) at GBs for each grain present in the photocurrent images of the two PSCs with different PCEs. The high-efficiency PSC exhibits an average PPE of approximately 5%, whereas the average value for the low-efficiency PSC is only around 1.4%. This comparison indicates that the role of GBs differs between high-efficiency and low-efficiency PSCs. This result is consistent with a previous proposition grounded in examinations of bare MHP films using atomic force microscopy (C-AFM) [23]. Our present research stands as the pioneering study to elucidate the positive impact of GBs through the direct visualization of photocurrent within operational PSCs.

### Built-in Fields at GBs and Charge Separation Dynamics

**Fig. 2:**
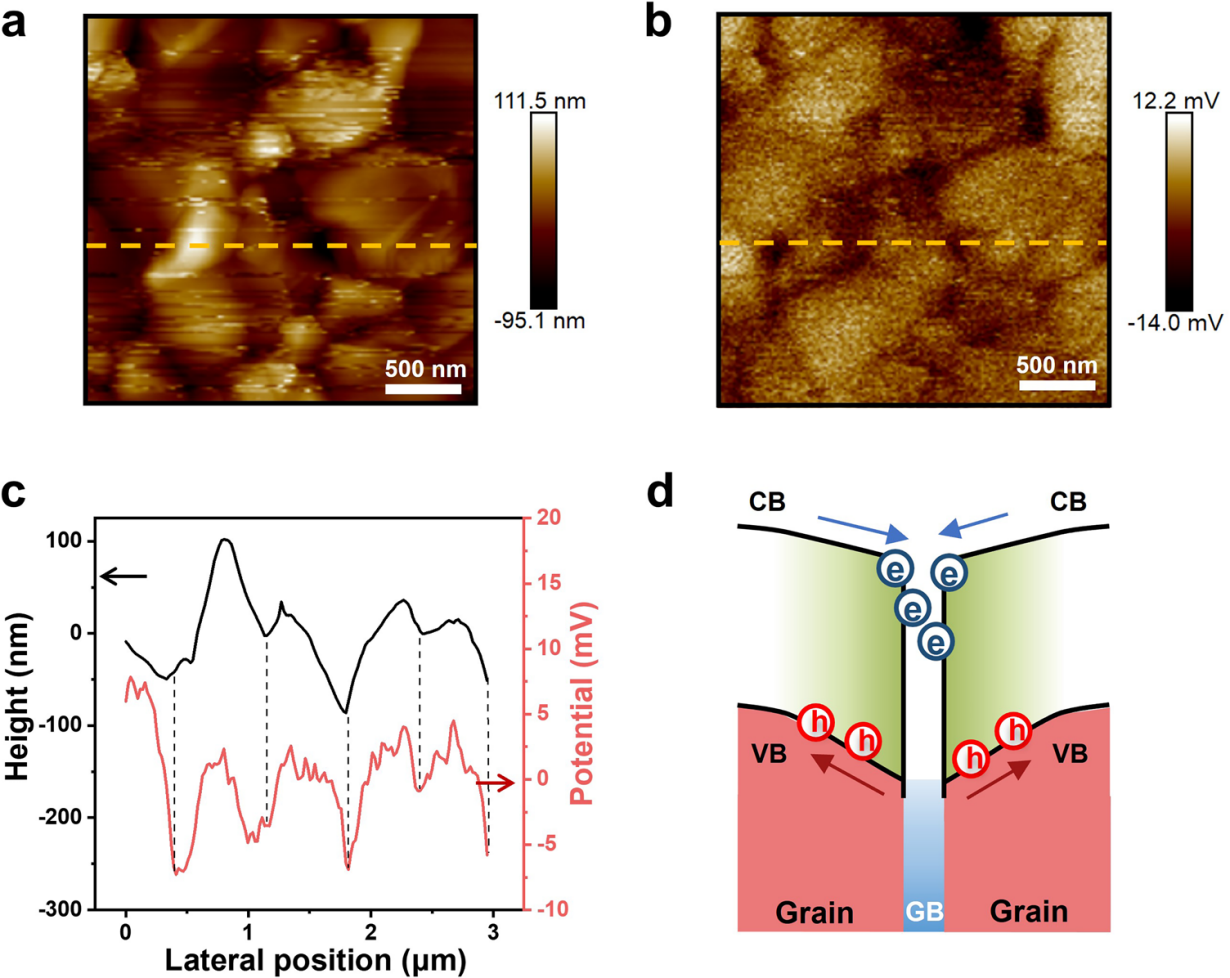
KPFM data for the bare perovskite film showing **a** the topography and **b** the respective CPD map. **c** One-dimensional line profiles of the topography and CPD amplitude along the yellow-dashed lines indicated in **a–b**. **d** Schematic illustration of the band alignment between GBs and GI. The built-in electric field induces a downward bending of the energy band at GB, resulting in electron accumulation

In addition to photocurrent, the local photovoltage distribution was also recorded for the operational PSCs. The high-efficiency PSC under open-circuit conditions also exhibits a notable enhancement in photovoltage at GBs (Fig. 1i). Figure 1j displays a pronounced anticorrelation between PL (Fig. S11) and photovoltage intensity at GBs. This indicates that, in comparison with the grains, the GBs are more effective in charge separation, resulting in the generation of a higher number of free carriers at the GBs. Previous studies have speculated that the beneficial impact of GBs on carrier kinetics may be linked to the existence of a built-in electric field at these boundaries, leading to a downward bending of the energy band and thereby aiding in the separation of electron–hole pairs [31]. We examined this speculation through KPFM measurements, which were carried out on an isolated high-quality MHP film (used in high-efficiency PSCs). The AFM topography image (Fig. 2a) distinctly differentiates between the grains and GBs. The surface contact potential difference (CPD) image acquired simultaneously in Fig. 2b sharply exhibits the contrast between the GBs and the grain interior (GI). The GBs show a smaller surface work function compared to the GI, generating a built-in electric field of 10^2^–10^3^ V cm^−1^ at GBs. This distinction can be further examined by aligning the surface topography with the corresponding one-dimensional surface potential fluctuation along the designated yellow-dashed line. As depicted in Fig. 2c, a sharp contrast in the surface potential between the GBs and GI is observed which is consistent with previous works [31]. The corresponding KPFM measurements provide an unequivocal understanding of the physical picture at the GBs of the perovskite polycrystalline film. As shown in Fig. 2d, the localized built-in potential results in electron attraction to the GBs and hole repulsion to the GI, thereby facilitating electron–hole separation.

To gain a comprehensive understanding of photogenerated carrier behavior in high-quality perovskite film, we employed TRPL measurements to probe the PL kinetics at both GB and GI in isolated perovskite film. Results reveal that the PL decay is faster at GBs than in GI, suggesting rapid carrier separation at GBs (Fig. S12a). To gain insight into this process, we utilize pump-probe transient absorption microscopy (TAM) to investigate the carrier kinetics at GBs and within GI.

**Fig. 3:**
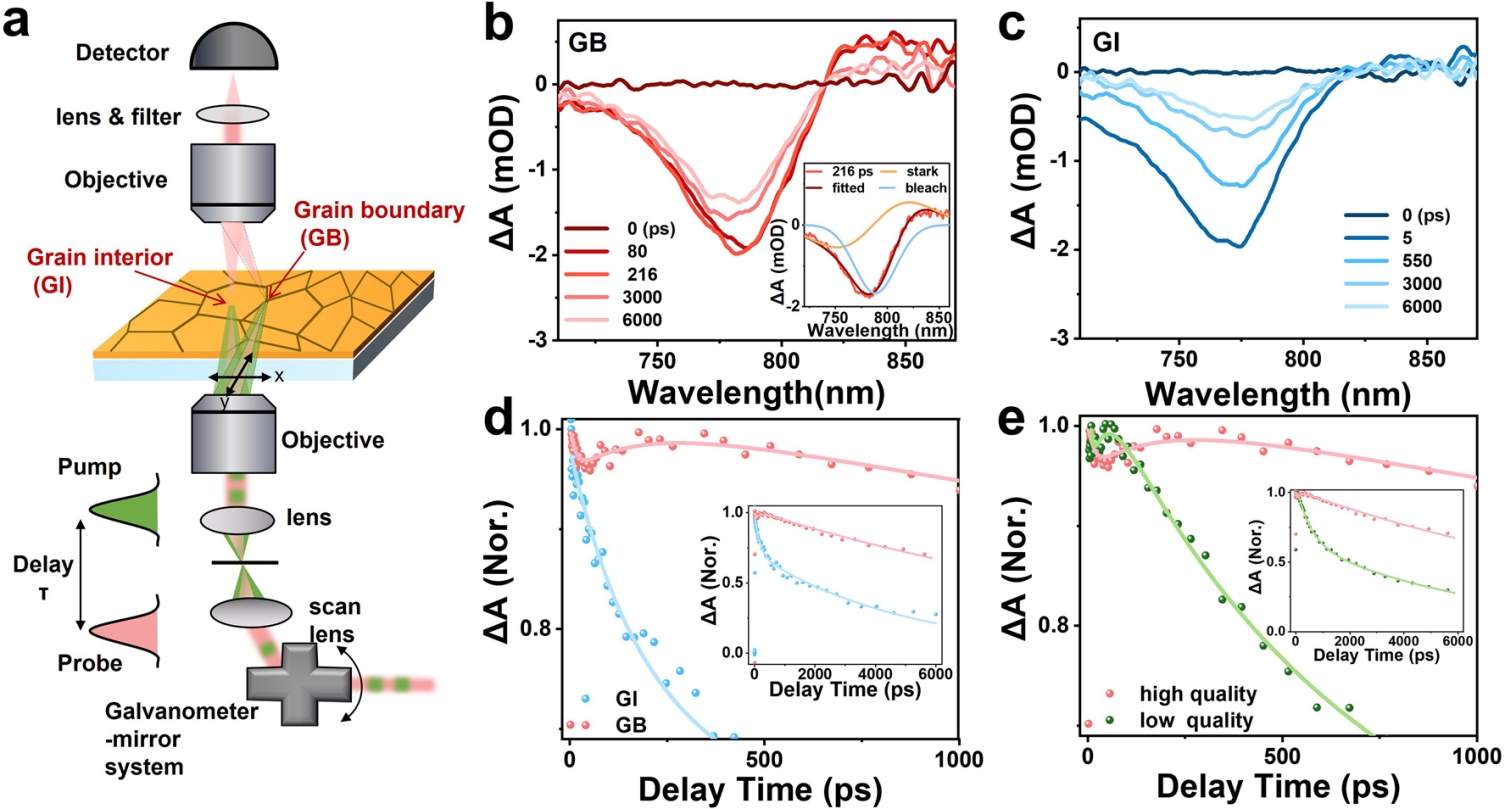
**a** Schematic illustration of pump-probe transient absorption microscopy. **b** TA spectra of high-quality perovskite film in GB, with an inset demonstrating its decomposition at 216 ps into Stark effect and bleach signals. **c** GI at indicated delay times under excitation intensity of 1.42 μJ cm^−2^ at 515 nm. **d** Comparison of the XB Kinetics at GB and GI reveals a distinctive 300 ps rise component in GBs. The solid lines are their exponential fits. **e** Comparison of the XB Kinetics at GBs in high- and low-quality perovskite films under excitation intensity of 1.42 μJ cm^−2^. The solid lines are their exponential fits. The inset represents the entire XB kinetics

Figure 3a shows the experimental setup of the pump-probe TAM, by which the transient absorption (TA) spectra at GB and GI are obtained. The TA spectra for both GB and GI in the high-quality MHP film are dominated by an exciton bleach (XB) feature due to the state-filling effect of band-edge electrons and holes (Fig. 3b, c) [39–41]. Meanwhile, the TA spectra display notable differences between GB and GI in the long wavelength region (> 800 nm). Specifically, the GB shows a much slower recovery process at the XB band and a positive signal above 800 nm. The latter implies the presence of a derivative-like signal above 800 nm which is usually observed as a characteristic of internal charge separation. This charge separation, driven by the built-in electric field at GB, can form a photoinduced electric field [39, 42] (also called a modulated field, opposite to the built-in field) and then lead to the occurrence of a derivative-like Stark signal by shifting band gap energy.

Stark signal in TA spectra, a signature of the photoinduced electric field, is widely reported in many semiconductor materials [43]. To accurately determine the kinetics of XB and electric field, we utilized a global-fitting procedure [44] to decompose the TA spectra at GB into two components: XB and stark signal (Inset in Figs. 3b and S13). The increase of the stark signal implies electron–hole separation, while its recovery indicates a reduction in the photoinduced electric field resulting from electron–hole recombination.

To unravel the carrier dynamics at GB and GI, we compare the XB kinetics in Fig. 3d, where a notable rising component in XB kinetics at the GB is observed. Because the electrons contribute a larger amplitude in the TA signal than the holes for perovskite materials [45], the rising component in the TA kinetics mainly reflects electron transfer from GI to GB driven by the build-in electric field at GB, and meanwhile, the holes transfer away from the GB. By global fitting the TA kinetics (see Supporting Information for the detail of fitting function), we determined an initial rapid decay and a rising process signifying an electron–hole separation time of ${\uptau }_{cs}\approx$ 166.9 ps (Table S1), aligning with the rising component in the stark signal at the GB (the photoinduced electric field) (Fig. S14). Meanwhile, XB kinetics at GB exhibit a markedly prolonged lifetime (14.5 ns) relative to those in GI (5.0 ns), demonstrating the presence of a long-lived electron–hole separation state at GB. This observation aligns with KPFM results and corresponds with the noted electron accumulation at GB in CH_3_NH_3_PbI_3_ (MAPbI_3_) perovskite films due to the electric field-induced downward band bending at GB [31]. This, in turn, facilitates electron–hole separation and enhancement of photocurrent in high-performance PSCs.

To further confirm the role of GB, we conducted TA measurements on a low-quality MHP film (Fig. S15), where the TA spectra of GBs exhibit a similar positive signal above 800 nm, indicating the ubiquity of a built-in electric field at GBs. We further isolated pure XB and stark signal using the global-fitting procedure (Fig. S16). A comparison of the XB kinetics at GBs between low-quality and high-quality perovskite films (Fig. 3e) indicates a much smaller amplitude in the rising component and a much faster decay process in low-quality perovskite films. This is because of the presence of a larger density of defects at GB in low-quality perovskite films, which thus weaken the build-in electric field-induced charge separation effect. By fitting the TA decay kinetics with a multi-exponential function, we identified the charge separation time of a fast defect-trapping process of 522.9 ps in the low-quality film (Table S2) which is consistent with the TRPL results (Fig. S1). These defects trap carriers, leading to carrier loss and consequently a reduction in photocurrent at GBs in low-performance PSCs.

### Dual Role of GBs in PSCs

**Fig. 4:**
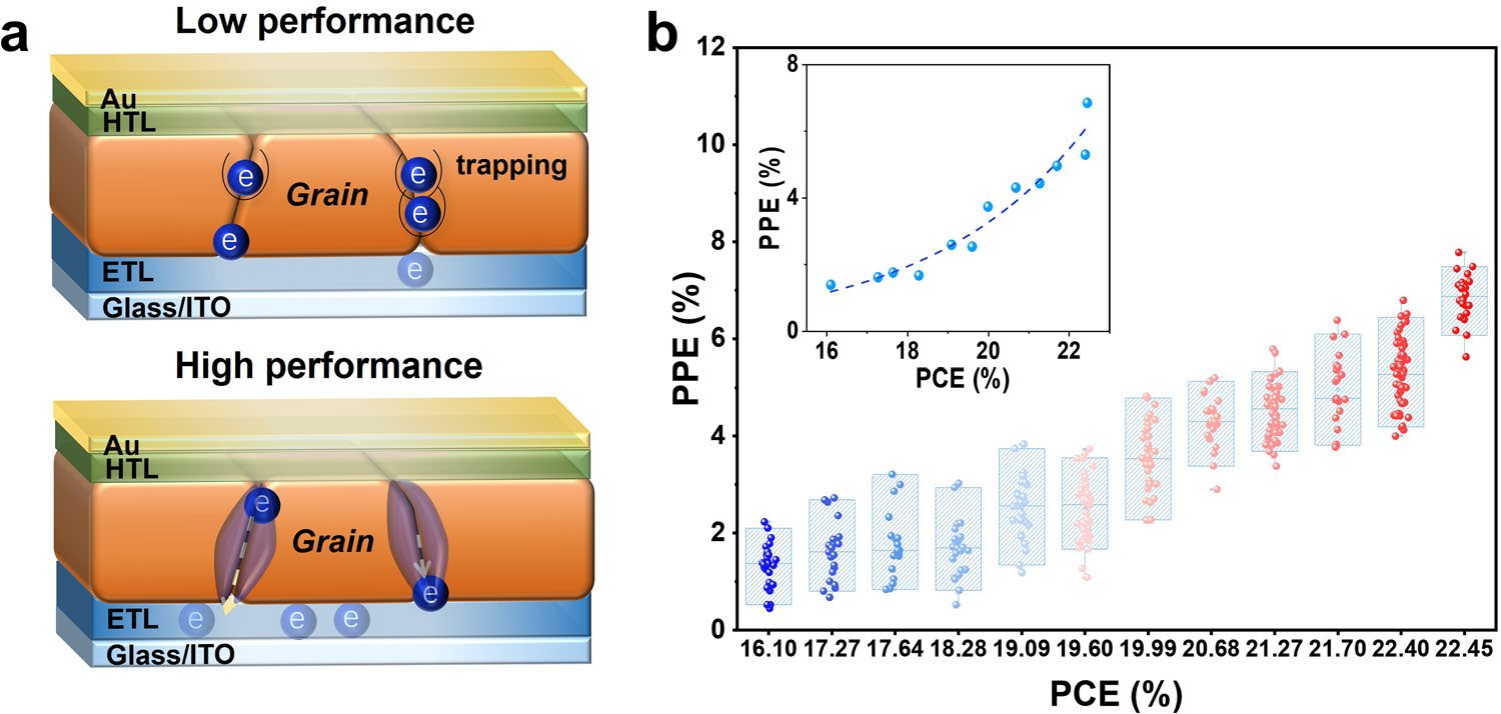
**a** Schematic illustration of the influence of GBs on electron behavior in both low- and high-performance PSCs. **b** Statistics of the correlation between the PPE at GBs and PCE in PSCs show that the PCE ranges from 16.10% to 22.45%. The inset represents an exponential growth correlation between the average PPE and the PCE of the solar cells

The above experimental results indicate that the GBs exhibit a dual nature in PSCs, similar to a double-edged sword. As schematically illustrated in Fig. 4a, the GBs serve as essential charge separation channels and play a positive role in high-performance PSCs by accumulating electrons and promoting charge collection to electrodes. On the other side, GBs are also ununiform microstructures where a high density of defects can be presented particularly in PSCs with a low-quality of perovskite film. In this case, the positive charge separation effect is significantly weakened by the defects. To quantify the contribution of GBs in PSCs of varying PCEs, we recorded the photocurrent in different PSCs with a PCE distribution from 22.45% to 16.10% (Fig. S17), which demonstrated an evident diminishing contrast in photocurrent intensity between GB and GI associated with a decrease in PCE. Figure 4b statistically analyzes the relationship between PCE and PPE, revealing a notable positive correlation that follows an exponential growth trend, thereby underlining the advantageous role of GBs in boosting PSC performance.

## Conclusions

In summary, this research provided meaningful insights into the dual role of GBs in operational PSCs and their impact on device performance. A notable photocurrent enhancement at GBs indicated that GBs function as pivotal electron–hole separation channels and play a positive role in high-performance PSCs. The presence of a built-in electric field at GBs, confirmed by KPFM and TA spectra measurements, effectively promotes charge separation, facilitating electron accumulation and subsequent charge extraction. On the other hand, the presence of high defect densities at GBs in low-performance PSCs leads to carrier loss and consequent reduction in photocurrent at GBs. This work clarified the role of GB in operational PSCs and highlights the potential for manipulating GB properties for the logical design of perovskite active layers and the development of high-performance and stable PSCs.

## Supplementary Information

Below is the link to the electronic supplementary material.

Supplementary file1 (DOCX 10466 KB)
