# Supplementary material for: Grain Boundaries Contribute to the Performance of Perovskite Solar Cells by Promoting Charge Separations
Source: Nanomicro Lett. 2025 Jun 4;17:285. doi: 10.1007/s40820-025-01795-0 (PMC12137848; doi:10.1007/s40820-025-01795-0)
Supplement: Supplementary file 1 — Supplementary file1 (DOCX 10466 KB) [file 40820_2025_1795_MOESM1_ESM.docx]

Supporting Information for

**Grain Boundaries Contribute to the Performance of Perovskite Solar Cells by Promoting Charge Separations**

Peng Xu^1,2,^†, Pengfei Wang^4,^†, Minhuan Wang^4,^†, Fengke Sun^2,5^, Jing Leng^2^, Yantao Shi^3^*, Shengye Jin^1,2^*, Wenming Tian^2^*

^1^ Department of Chemical Physics, University of Science and Technology of China, Hefei 230026, P. R. China

^2^ State Key Laboratory of Chemical Reaction Dynamics, Dalian Institute of Chemical Physics, Chinese Academy of Sciences, Dalian 116023, P. R. China

^3^ State Key Laboratory of Fine Chemicals, School of Chemistry, Frontier Science Center for Smart Materials, Dalian University of Technology, Dalian 116024, P. R. China

^4^ Key Laboratory of Materials Modification by Laser, Ion and Electron Beams (Ministry of Education), School of Physics, Dalian University of Technology, Dalian 116024, P. R. China

^5^ University of Chinese Academy of Sciences, Beijing 100049, P. R. China

† Peng Xu, Pengfei Wang, and Minhuan Wang contributed equally to this work.

*Corresponding authors. E-mail: [shiyantao@dlut.edu.cn](mailto:shiyantao@dlut.edu.cn) (Yantao Shi); [sjin@dicp.ac.cn](mailto:sjin@dicp.ac.cn) (Shengye Jin); [tianwm@dicp.ac.cn](mailto:tianwm@dicp.ac.cn) (Wenming Tian)

S1 Experimental Section

**S1.1 Materials**

The SnO_2_ colloid (tin (IV) oxide) precursor (15 wt% in H_2_O colloidal dispersion) was obtained from Alfa Aesar. Ndimethylformamide (DMF), dimethyl sulfoxide (DMSO, 99.9%), acetonitrile (ACN, 99.8%), methylbenzene (99.9%), chlorobenzene (CB, 99.8%) and Bis (trifluoromethane) sulfonimide lithium salt (Li-TFSI, 99.95% trace metals basis) were obtained from Sigma-Aldrich. Lead iodide PbI_2_ (>99.999%) and Formamidinium iodide (FAI) (99.8%) were obtained from Advanced Election Technology Co., Ltd. Methylammonium bromide (MABr) (99.9%) and methylammonium chloride (MACl) (99.9%) were purchased from Xi’an Polymer Light Technology. 2,2′,7,7′-tetrakis (N,Ndip-methoxyphenylamine)-9,9′- spirobifluorene (Spiro-OMeTAD, 99.8%) was purchased from Borun New Material Technology. Au and Ag were purchased from ZhongNuo Advanced Material (Beijing) Technology Co., Ltd. All the chemicals were used as received without further treatment

**S1.2 Device fabrication**

The ultra-thin ITO (0.15 mm) substrates were washed with deionized water, ethanol and isopropanol successively and were dried under nitrogen flow. After 15 minutes of UV-ozone treatment, the SnO_2_ colloidal dispersion was diluted to 2.5 wt% with deionized water. Subsequently, the prepared SnO_2_ solution was spun onto the blow-dried ultra-thin ITO substrate with 3000 rpm for 30 s and heated at 150 °C for 30 minutes in ambient air to prepare the electron-transport layer (ETL). Then, reserved 5 min UV-ozone treatment. and transferred to the N_2_ glove box. The (FAPbI_3_)_0.95_(MAPbBr_3_)_0.05_ precursor solution was prepared by adding FAI(274.46 mg), PbI_2_(735.77 mg), MABr(9.41mg), of PbBr_2_(30.83mg) and MACl(30.83mg) into 1.2mL of a mixed solvent of DMF and DMSO (7:1 by volume) stirred at room temperature for 6 h. To prepare the metal halide perovskite (MHP) film, 50 μL of above perovskite precursor was spread on the ITO/SnO_2_ substrates and spun by a two-stage spin-coating process (1000 rpm for 10 s and 5000 rpm for 30 s). During the second spin coating stage, 150 μL of methylbenzene was continuously dripped on the spinning substrate 15s prior the end of the program. The films were then transferred to a hot plate and annealed at 120 °C for 5and 20 minutes. Precursor solution of HTL was prepared by dissolving 72.3mg spiro-MeOTAD, 28.8μL 4-tert-butylpyridine, 17.5μL lithium bis (trifluoromethylsulphonyl) imide acetonitrile solution (520 mg mL^-1^), 20 μL FK209 acetonitrile solution (300 mg mL^-1^) into 1 mL CB. The HTL solution was then deposited on top of the perovskite layer by spin coating at 3,000 rpm for 30 s. Finally, an 80 nm Au electrode was deposited by thermal evaporation on top of the HTL.

**S1.3 Preparation of high-quality and low-quality (FAPbI_3_)_0.95_(MAPbBr_3_)_0.05_ perovskites thin film**

The distinction between high-quality and low-quality perovskite films in this study was achieved solely by modulating the annealing time, while all other fabrication parameters remained identical. Both films were prepared using the same precursor solution and spin-coating conditions. The (FAPbI_3_)_0.95_(MAPbBr_3_)_0.05_ precursor solution was prepared by adding FAI(274.46 mg), PbI_2_(735.77 mg), MABr(9.41mg), of PbBr_2_(30.83mg) and MACl(30.83mg) into 1.2mL of a mixed solvent of DMF and DMSO (7:1 by volume) stirred at room temperature for 6 h. 50 μL of above perovskite precursor was spread on the glass substrates and spun by a two-stage spin-coating process (1000 rpm for 10 s and 5000 rpm for 30 s). During the second spin coating stage, 150 μL of methylbenzene was continuously dripped on the spinning substrate 15s prior the end of the program. High-quality films were synthesized by annealing the sample at 120°C for 20 minutes in a nitrogen atmosphere. In contrast, low-quality films resulted from a shorter annealing duration of 5 minutes.

**S1.4 Materials characterization**

The microstructures of the thin films were observed using a field-emission SEM (JSM-7610F Plus, Hitachi, Japan). The roughness images were tested using an atomic force microscope (AFM, NT-MDT Spectrum Instruments, Ntegra Prima, Russia) in the contact mode. The surface potential of the thin films was measured by the KPFM modules in the AFM instrument. The PL spectra were obtained by a monochromator (SpectraPro-HRS-300, Princeton Instruments, USA) coupled with a charge-coupled device (CCD) camera (PIXIS 100, Princeton Instruments, USA). The crystalline phase and crystallinity of MHP film were collected by X-ray diffractometer (XRD, Rigaku, Smart Lab 9 kw, Japan) with Cu Kα radiation.

**S1.5 Solar cell performance**

The J-V characteristics of PSCs were measured by a source meter (2450, Keithley, USA) at the scan speed of 100 mV s^-1^ under AM 1.5G one-sun illumination (100 mW cm^-2^) generated by a solar simulator (Sol3A Class AAA, Oriel, Newport, USA). The intensity was calibrated using an ISO-17025 standard calibrated reference silicon cell (91150V-KG3, Newport, USA). The active device area of PSCs during the measurements was 0.049 cm^2^ as defined by a metal mask. Steady-state current/PCE outputs were measured using the 2450 Source Meter (Keithley, USA) at voltages determined from the MPPs of the reverse-scan J-V curves. All performance tests are carried out in ambient air at room temperature.

**S1.6 Transient absorption (TA) spectra measurement**

The femtosecond TA spectrometer is based on a Yb:KGW laser system (1030 nm,100 kHz; Light Conversion Ltd.), nonlinear frequency mixing techniques and the Femto-TA100 spectrometer (Time-Tech Spectra LLC). Briefly, the fundamental output at 1030 nm was split in two parts. One part was introduced to a noncollinear optical parametric amplifier to generate a certain wavelength for pump beam. The other was focused onto a YAG crystal to generate continuum white light (550–950nm) as probe beam. The probe beam was focused with a parabolic reflector onto the sample and the transmitted probe beam was then collimated and focused into a fiber coupled spectrometer with CMOS sensors. The intensity of the pump pulse used in the experiment was controlled by a variable neutral-density filter wheel. The delay between the pump and probe pulses was controlled by a motorized delay stage. The pump pulses were chopped by a synchronized chopper at 5 KHz and the absorbance change was calculated with two adjacent probe pulses (pump-blocked and pump unblocked).
Transient absorption spectroscopy in micro-nano space was collected by a home-built transient absorption microscopy (TAM) system. The schematic is shown in Figure. 3a. Briefly, the fundamental output at 1030 nm was split into two parts. One part was introduced to a noncollinear optical parametric amplifier to provide the pump (515nm). The other was focused onto a YAG crystal to generate continuum white light (550–950nm) as probe beam. Both the pump and probe beams were spatially filtered. Both the pump and probe beams were focused onto the sample by an objective lens(100X, NA = 1.45, Olympus UPlanSApo). The probe beam was then collected by another objective lens(100X, NA = 1.45, Olympus UPlanSApo) and the transmitted probe beam was then collimated and focused into a fiber-coupled spectrometer with CMOS sensors. The intensity of the pump pulse used in the experiment was controlled by a variable neutral-density filter wheel. The delay between the pump and probe pulses was controlled by a motorized delay stage. The pump pulses were chopped by a synchronized chopper at 5 KHz and the absorbance change was calculated with two adjacent probe pulses (pump blocked and pump unblocked). A pair of Galvanometer mirror (Thorlabs GVS002) was used to adjust the location of probe beam and pump beam in space to obtain transient absorption spectroscopy in micro-nano space.

This experimental design intentionally creates a spatially uniform excitation by ensuring the pump beam (4 μm) completely overlaps and exceeds the size of the probe beam (1 μm). By confining the probed area entirely within the uniformly photoexcited region, carrier density gradients are eliminated at the probe boundaries. The absence of such gradients inherently suppresses diffusion-driven carrier motion, as diffusion requires a concentration difference to occur [S1].

**S1.7 Laser-scanned confocal PL imaging and photocurrent & photovoltage imaging measurement**

The PL and photocurrent imaging measurements are performed on a home-built laser-scanned and time-resolved PL microscopy coupled with a photocurrent detection module. The schematic is shown in Figure 1a. For PL measurements, a pulse laser with wavelength of 405 nm (NPIL1-040-40FC, NKT Photonics, Denmark) is focused on the sample through a 100X oil objective lens (NA = 1.45, Olympus UPlanSApo). The laser intensity at samples is adjusted by a neutral density filter, and measured with a power meter (PM100D S130VC, Thorlabs, USA). The PL signal is collected using a high-speed detector (MPD, PicoQuant, Germany) with a 710 nm and 420 nm long-pass filter. Besides, a 25 μm pinhole size is chosen during the experiments. For photocurrent measurements, a CW laser with wavelength of 405 nm is coupled to the system and the photocurrent & photovoltage are detected by using a Keithley 6485 picoammeter(Tektronix, USA) and Keithley 4200A-SCS parameter analyzer (Tektronix, USA). The photocurrent and photovoltage images are obtained by scanning the laser spot and recording the photocurrent during the scanning of each point on the perovskite solar cell.

In this experiment, 405 nm were selected as excited light, and the energy density to excite the sample was set to 7.64 W cm^-2^ (1.4 ×10^16^ cm^-3^).

**S1.8 Power-dependent photocurrent measurements**

Photocurrent image measurement was conducted at different power densities and the results were shown in Figure S8. Generally, for all power used in the experiment, exhibited similar photocurrent trends in GBs. We obtain n ≈1.4 * 10^16^ cm^-3^ , which is well below the Mott density (~10¹⁸ cm⁻³), ensuring that the measured photocurrent trends reflect intrinsic GB properties.

**S1.9 ​​Continuous reflection interface sampling and positioning (CRISP) continuous autofocus system**

The ​​Continuous Reflection Interface Sampling and Positioning (CRISP) continuous autofocus system​​ (Applied Scientific Instrumentation, Inc.) is integrated into our photocurrent mapping setup to ensure exceptional focal stability. This system utilizes a ​​970 nm infrared (IR) LED​​ to monitor the perovskite film’s ​​bottom interface​​via real-time optical feedback. The IR LED light is reflected by the dichroic beam splitter (DMSP900R, thorlab​​) into the microscope objective and visible light passes through the dichroic beam splitter.

**S1.10 Calculation of percentage of photocurrent enhancement (PPE) at grain boundary (GB)**

Photocurrent image provides the photocurrent intensity (*I*) at points within the micro-nano space. *I_GB_* is the average photocurrent intensity of two adjacent pixels at the GB in the photocurrent image. *I_GI_* denotes the average photocurrent intensity within the grain that is surrounded by the grain boundary.

$$\begin{aligned} PPE=\frac{I_{GB}-I_{GI}}{I_{GI}}\#\left( S1 \right) \end{aligned}$$

By calculating the PPE of devices with varying power conversion efficiencies (PCE), we can establish the relationship between PPE and PCE in Figure 1h and Figure 4b.”

**S1.11 Decomposing the transient absorption (TA) spectra into bleach, Stark effect signals**

We performed spectra decomposition using a global fit based on the least-squares fitting method. To apply this approach, we measured the TA spectra, ΔA(*λ*, *T_d_*), as a function of wavelength (*λ*) at various delay times (*T_d_*). The TA spectrum is modeled as a linear combination of the following components: Stark effect signal (*S(λ)*), and bleach signal (*B(λ)*).

$$\begin{aligned} \Delta A\left( \lambda, T_{d} \right)=C_{1}\left( T_{d} \right)*S\left( \lambda\right)+C_{2}\left( T_{d} \right)*B\left( \lambda\right)\#\left( S2 \right) \end{aligned}$$

Where, C_1_ and C_2_ are functions of T_d_, while the bandshapes of *(S(λ)), (B(λ)).*

$$\begin{aligned} B\left( \lambda\right)=-\sum_{n} \left( {a_{n}\cdot e}^{-\frac{\left( \lambda-c_{n} \right)^{2}}{2{\omega_{n}}^{2}}} \right)\#\left( S3 \right) \end{aligned}$$

Where a_n_, c_n_, and ω_n_ are float parameters being optimized during the global fits. The shape of the Stark effect signal, *S(λ)*, is modeled as the first-order derivatives of multi-Gaussian peaks.

$$\begin{aligned} G\left( \lambda\right)=\sum_{n} \left( {a_{n}\cdot e}^{-\frac{\left( \lambda-c_{n} \right)^{2}}{2{\omega_{n}}^{2}}} \right)\#\left( S4 \right) \end{aligned}$$

$$\begin{aligned} S\left( \lambda\right)=G^{'}\left( \lambda\right)\#\left( S5 \right) \end{aligned}$$

Where an, c_n_, and ω_n_ are also float parameters being optimized during the global fits. G'(λ) represents the first derivatives of G(λ). We determine the values of C_1_ and C_2_ (in Equation (S2)) and the bandshapes of *S(λ)* and *B(λ)* through the global fitting, minimizing the difference between the experimentally measured ΔA (*λ*, *T_d_*) and the values calculated by Equation (S2) using a least-squares method. After the global fits, we can extract the bleach and Stark components.

$$\begin{aligned} {\Delta A}_{\mathrm{Stark}}\left( \lambda, T_{d} \right)= C_{1}(T_{d})*S\left( \lambda\right)\#\left( S6 \right) \end{aligned}$$

$$\begin{aligned} {\Delta A}_{\mathrm{bleach}}\left( \lambda, T_{d} \right)= C_{2}(T_{d})*B\left( \lambda\right)\#\left( S7 \right) \end{aligned}$$

Figure S7 shows the corresponding data for different *T_d_* in high-quality film, whereas Figure S10 presents similar data for low-quality film. Through this methodology, the two components are effectively separated.

**S1.12 Global fitting of the TA kinetics**

We performed a global fitting on three sets of decay kinetics to determine the electron-hole separation time in high-quality film. Two of the datasets, corresponding to the bleach and stark decay kinetics at GB, were fitted using a three-exponential function, while the third dataset, which involved bleach decay kinetics at GI, was fitted with a bi-exponential decay function. The models used for fitting are as follows:

For the first two datasets:

$$\begin{aligned} y_{i}\left( x \right)=A_{i1}e^{\frac{-x}{t_{1}}}+A_{i2}e^{\frac{-x}{t_{2}}}+A_{i3}e^{\frac{-x}{t_{3i}}} for i=1,2 \left( S8 \right) \end{aligned}$$

For the third dataset:

$$\begin{aligned} y_{3}\left( x \right)=A_{31}e^{\frac{-x}{t_{2}}}+A_{32}e^{\frac{-x}{t_{33}}} \left( S9 \right) \end{aligned}$$

Where *t_1_* and *t_2_* respectively represent the time constants of hole and electron transfer. *t_3i_* respectively represent their respective decay time constants. The amplitudes, *A_ij_ ,*are also float parameters being optimized during the global fits.

To minimize the sum of the squared residuals for all datasets, the following function is constructed:

$$\begin{aligned} \chi^{2}=\sum_{i=1}^{2} \sum_{j=1}^{ni} \left( yi\left( x_{j} \right)-\left( A_{i1}e^{\frac{{-x}_{j}}{t_{1}}}+A_{i2}e^{\frac{{-x}_{j}}{t_{2}}}+A_{i3}e^{\frac{{-x}_{j}}{t_{3i}}} \right) \right)^{2}+ \\ \sum_{k=1}^{n_{3}} {(y_{3}\left( x_{k} \right)-\left( A_{31}e^{\frac{{-x}_{k}}{t_{2}}}+A_{32}e^{\frac{{-x}_{k}}{t_{33}}} \right)}^{2} \#\left( S10 \right) \end{aligned}$$

Where *y_i_( x_j_ )* represents the data for the *i-th* dataset at time *t_j_*.

We determine the values of *t_1_* and *t_2_* (in Equation (10)) through the global fitting, minimizing the total residual $\chi^{2}$ calculated by Equation (10) using the Levenberg-Marquardt algorithm for the nonlinear least squares fitting process. After the global fits, we can obtain the time constants of electron-hole separation.

**S2 Supplementary Figures and Tables**


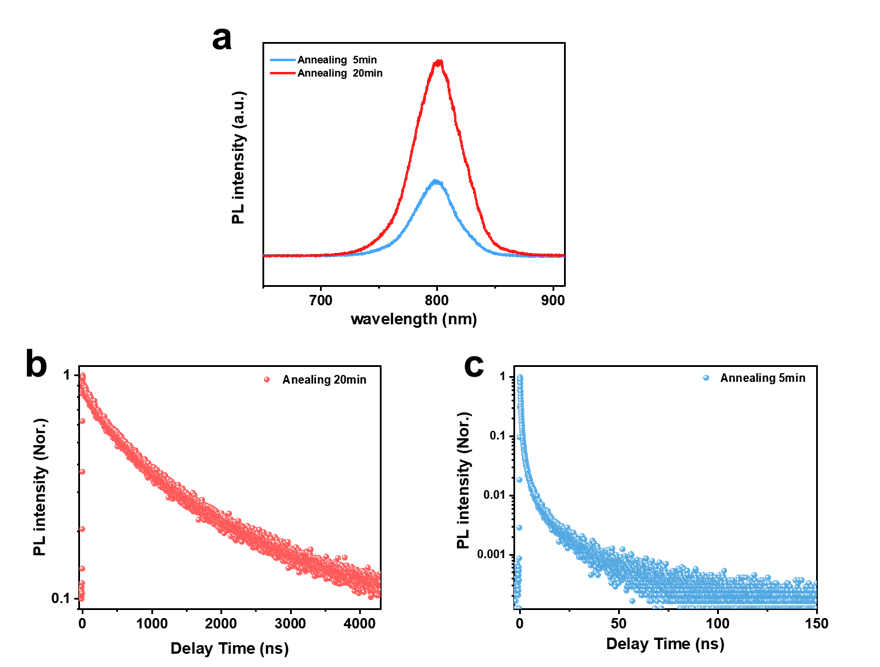


**Fig. S1** (**a**) PL spectra of MHP film for annealing 20 min (red) and annealing 5 min (blue) under laser excitation at 405nm with other preparation conditions unchanged. TRPL kinetics of MHP film for annealing 20 min (**b**) and annealing 5 min (**c**)


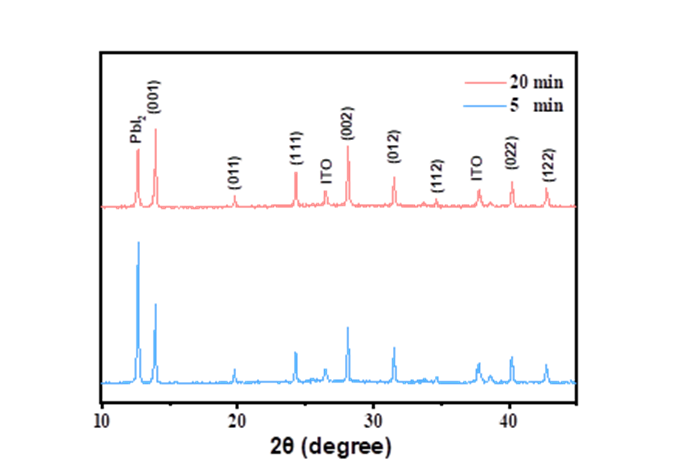


**Fig. S2** Comparison of XRD patterns of perovskite films with annealing 5 min and 20 min

**Fig. S3** SEM top-view images of perovskite films annealed for 5 min (**a**) and 20 min (**b**), respectively, combined with their corresponding grain size distribution histograms

**Fig. S4** Cross-section SEM image of high-quality film (**a**) and low-quality film (**b**)

**Fig. S5** Photocurrent profile extracted from the cross line (**a**) in photocurrent image of PSCs (**b**). ~500nm spatial resolution of the photocurrent mapping is estimated from the sharp drop. PL profile extracted from the cross line (**c**) in PL image of PSCs (**d**). ~300nm spatial resolution of the PL mapping is estimated from the sharp drop


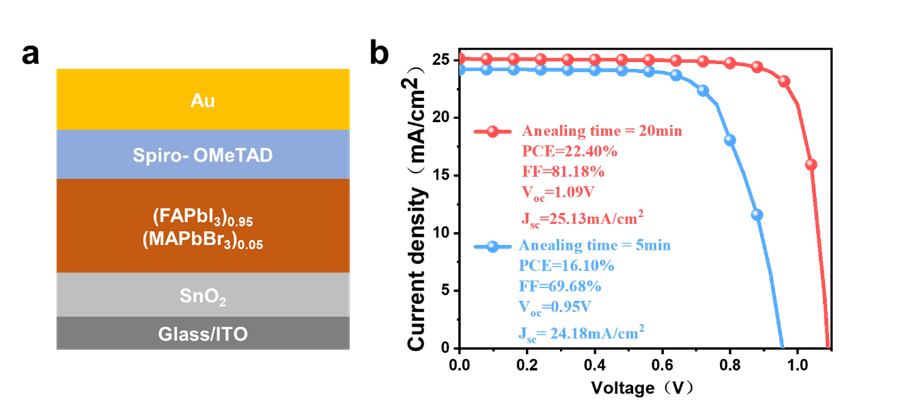


**Fig. S6** (**a**) Schematic device architecture of the n-i-p PSCs. (**b**) J–V plots for the performance of the PSCs device annealing for 20 min and 5 min, from 1.2 to 0 V as backward sweep. Detailed photovoltaic performance parameters are inset

**Fig. S7** (**a**) PL image (left) and bright-field image (right) at the same area. (**b**) We selected four regions for detailed analysis and performed a direct comparison between photoluminescence (PL) images and bright-field microscopy. The results revealed that areas with weak PL intensity corresponded to the positions of GBs identified in bright-field images, particularly in numbered large grains where this correlation was more pronounced


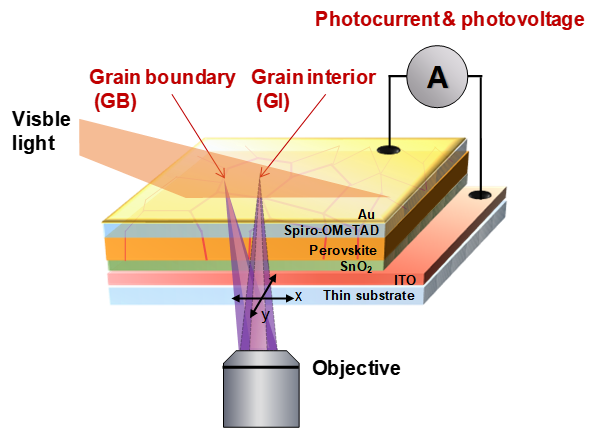


**Fig. S8** Schematic illustration of the experiment: A halogen lamp is used as a large-area excitation source (100 μw cm^-2^) to simulate the device's operating conditions while a 405 nm continuous laser performs confocal scanning


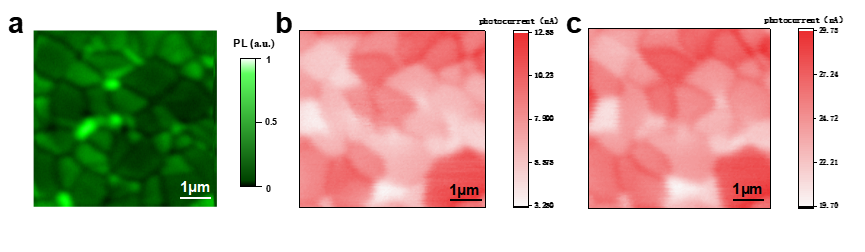


**Fig. S9** (**a**) Confocal PL intensity image of the PSC. (**b**) The photocurrent image of the same area as in (**a**) under non-operating conditions with only the 405 nm laser. (**c**) The photocurrent image of the same area as in (**a**) under operating conditions with both continuous white light and 405 nm laser. There is no discernible difference in the local photocurrent images under both operating and non-operating conditions (with or without continuous white light), except for the photocurrent intensity, which is affected by the additional continuous white light illumination.

**Fig. S10** Photocurrent imaging of a perovskites solar cell with efficiency equal to 22.38% under 405nm excitation at (**a**) 2.29 W/cm^2^ (**b**) 2.80 W/cm^2^ (**c**) 4.84 W/cm^2^ (**d**) 7.64 W/cm^2^ (**e**) 10.19 W/cm^2^, and (**f**) PL imaging at the same area


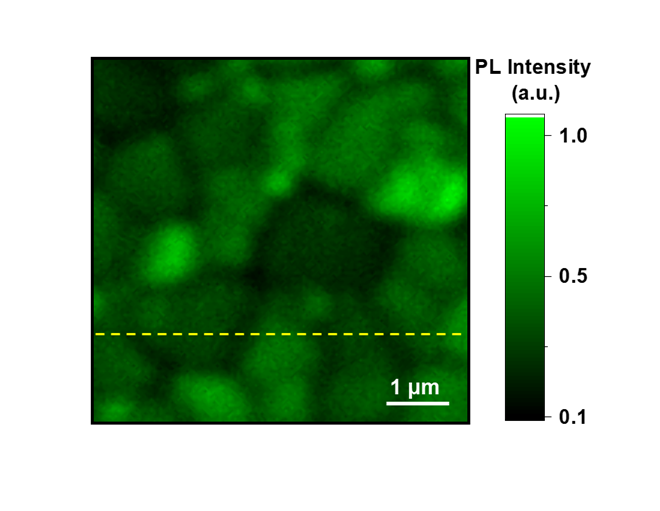


**Fig. S11** Confocal PL intensity image of a PSC with PCE of 22.29% under 405 excitation on the same area as **Fig.** 1(**i**)


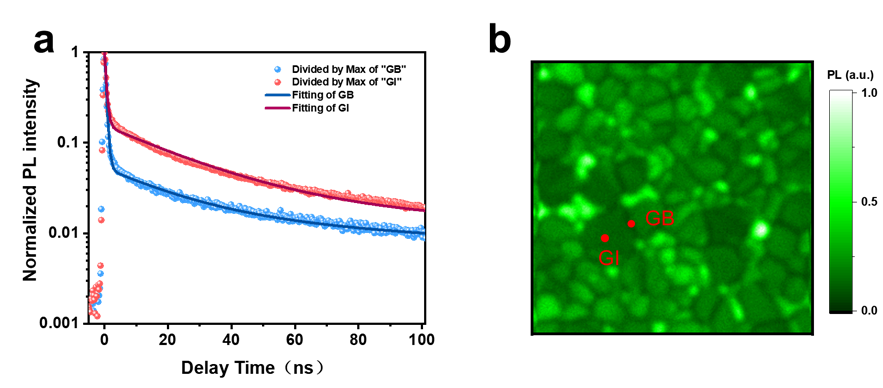


**Fig. S12** (**a**) Comparison of the TRPL kinetics at GB and GI as indicated in PL intensity image in (**b**)


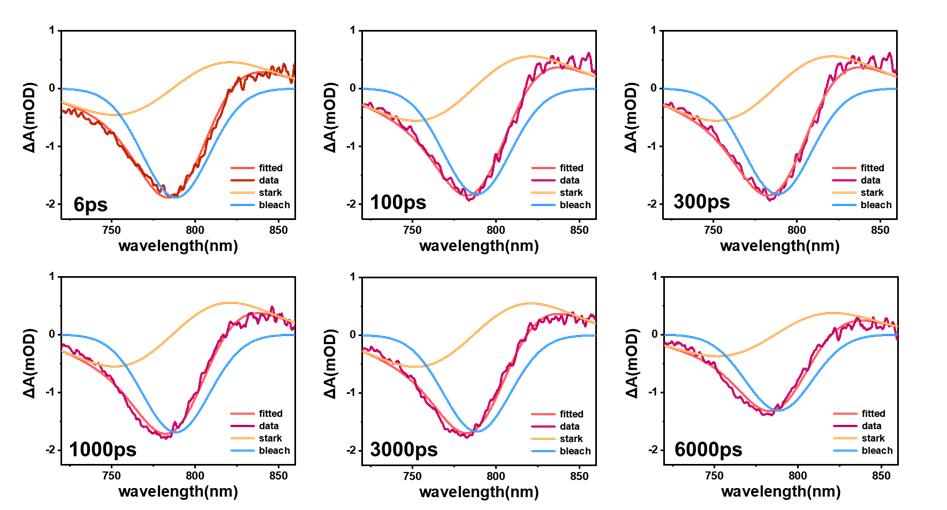


**Fig. S13** The multipeak Gaussian fitting of MHP of annealing for 20 min. The bleach signal is fitted with a Gaussian function, and the stark signal is fitted with the first-order derivative of the Gaussian function


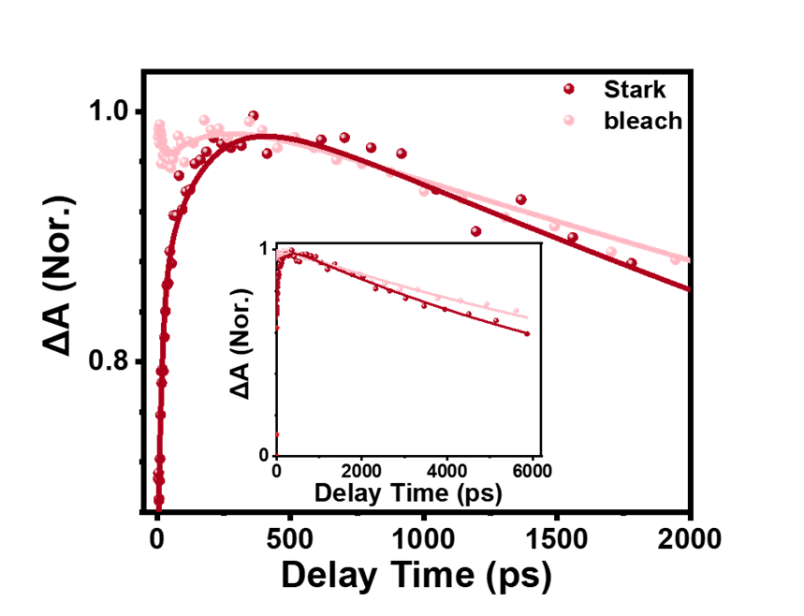


**Fig. S14** Comparison of the bleach and stark kinetics of MHP film annealed for 20 minutes. The inset represents the entire bleach and stark kinetics. Global fitting reveals two distinct time constants: τ₁= 15.0 ps and τ_2_ (166.9 ps). The τ₁ component reflects ​​hole migration away from GBs​​, while τ2 corresponds to ​​electron migration from grain interiors to GBs​​


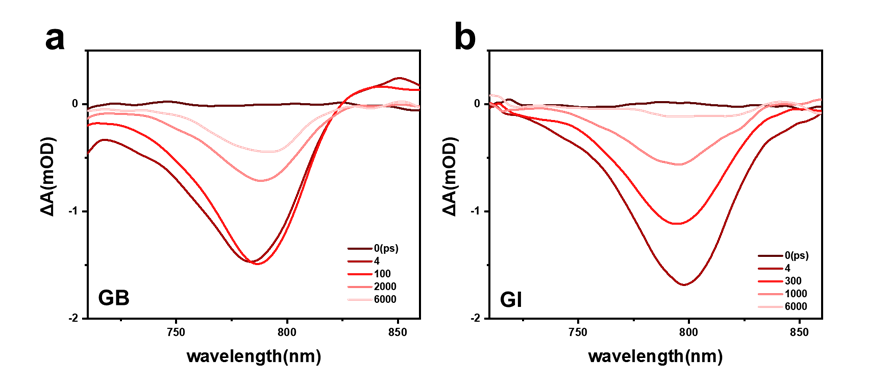


**Fig. S15** (**a**) TA spectra of low-quality perovskite film in GB and GI (**b**) at indicated delay times under excitation intensity of 1.42 μJ/cm^2^ at 515 nm


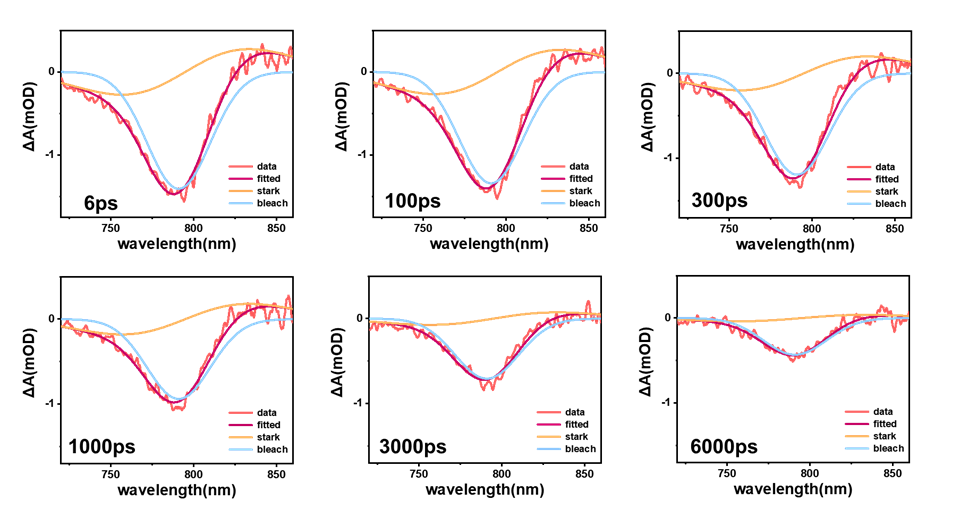


**Fig. S16** The multipeak Gaussian fitting of MHP annealed for 5 min. The bleach signal is fitted with a Gaussian function, and the stark signal is fitted with the first-order derivative of the Gaussian function


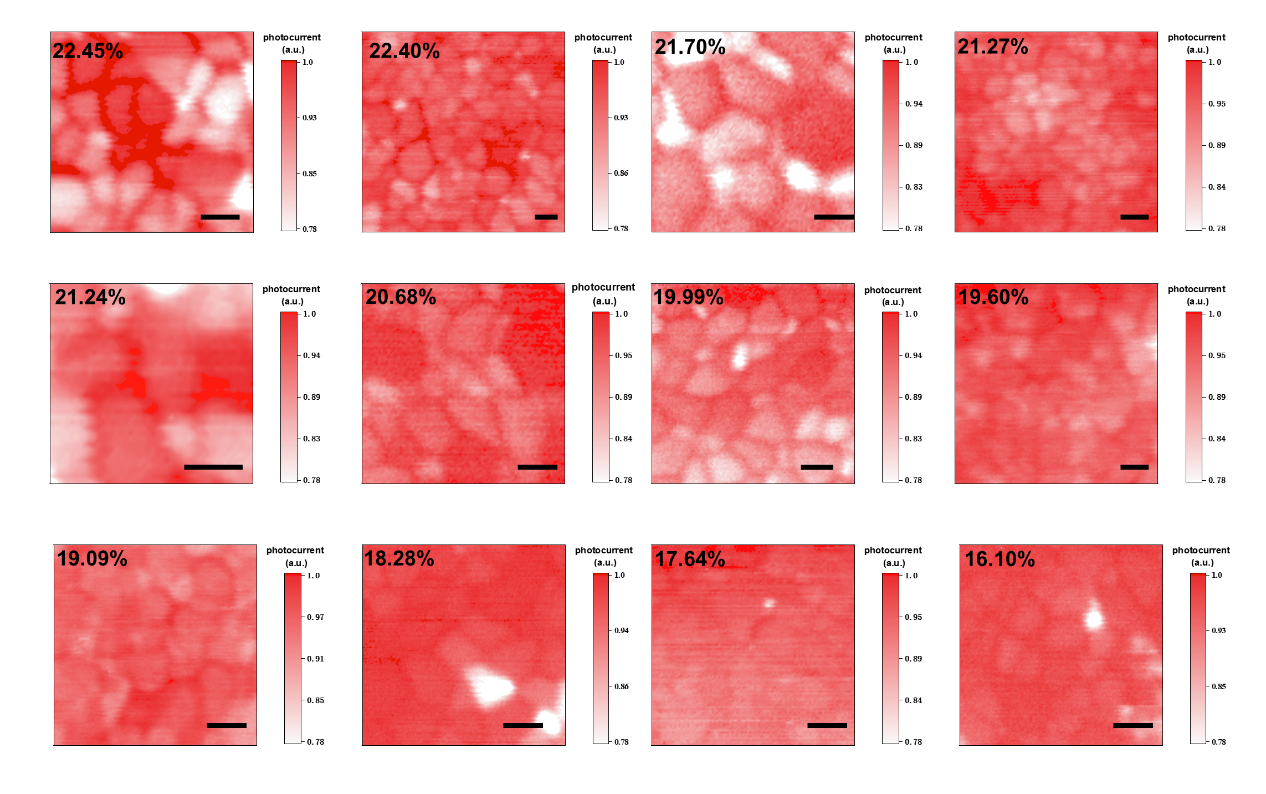


**Fig. S17** The normalized photocurrent images of PSCs with PCE distribution from 22.45% to 16.10%. The scale bars are 1μm

**Table S1** The fitting parameters for TA decay kinetics depicted in Figs. 3d, e and S14 of high-quality films

| **MHP film** | **τ_1_(A_1_) / ps** | **τ_2_(A_2_) / ps** | **τ_3_(A_3_) / ps** |
| --- | --- | --- | --- |
| **XB @GB** | 15.0  (0.03) | 166.90  (-0.06) | 14495.1  (1.02) |
| **Stark @GB** | 15.0  (-0.21) | 166.90  (-0.18) | 10632.4  (1.03) |
| **XB @GI** | ---- | 166.9  (0.26) | 4967.7  (0.71) |

**Table S2** The fitting parameters for TA decay kinetics depicted in Fig. 3e of low-quality films

| **MHP film** | **τ_1_(A_1_) / ps** | **τ_2_(A_2_) / ps** | **τ_3_(A_3_) / ps** | **τ_4_(A_4_) / ps** |
| --- | --- | --- | --- | --- |
| **XB @GB** | 5.4  (0.001) | 35.5  (-0.06) | 6896.8 (0.65) | 522.9 (0.39) |

**Supplementary References**

1. K. Omae, Y. Kawakami, S. Fujita,Y. Narukawa, T. Mukai, Effects of internal electrical field on transient absorption in In_𝑥_Ga_1−𝑥_N thin layers and quantum wells with different thickness by pump and probe spectroscopy. Phys. Rev. B **68**, 085303 (2003) <https://doi.org/10.1103/PhysRevB.68.0853038>
